# Underwater Acoustic Sensor Networks Node Localization Based on Compressive Sensing in Water Hydrology

**DOI:** 10.3390/s19204552

**Published:** 2019-10-19

**Authors:** Sen Wang, Yun Lin, Hongxu Tao, Pradip Kumar Sharma, Jin Wang

**Affiliations:** 1 College of Information and Communication Engineering, Harbin Engineering University, Harbin 150001, China; wangsen@hrbeu.edu.cn (S.W.); linyun@hrbeu.edu.cn (Y.L.); 2013082319@hrbeu.edu.cn (H.T.); 2 Department of Multimedia Engineering, Dongguk University, Seoul 04620, Korea; pradip.academic@gmail.com; 3 Hunan Provincial Key Laboratory of Intelligent Processing of Big Data on Transportation, School of Computer & Communication Engineering, Changsha University of Science & Technology, Changsha 410004, China; 4 School of Information Science and Engineering, Fujian University of Technology, Fujian 350118, China

**Keywords:** water hydrology, groundwater environment monitoring, node localization, compressive sensing

## Abstract

Groundwater is an important source of human activities, agriculture and industry. Underwater Acoustic Sensor Networks (UASNs) is one of the important technologies for marine environmental monitoring. Therefore, it is of great significance to study the node self- localization technology of underwater acoustic sensor network. This paper mainly studies the node localization algorithm based on range-free. In order to save cost and energy consumption, only a small number of sensing nodes in sensor networks usually know their own location. How to locate all nodes accurately through these few nodes is the focus of our research. In this paper, combined with the compressive sensing algorithm, a range-free node localization algorithm based on node hop information is proposed. Aiming at the problem that connection information collected by the algorithm is an integer, the hop is modified to further improve the localization performance. The simulation analysis shows that the improved algorithm is effective to improve the localization accuracy without additional cost and energy consumption compared with the traditional method.

## 1. Introduction

In recent years, with the development of remote sensing technology, some intelligent computing paradigms have been applied gradually in water hydrology and related research problems [1]. Groundwater is a very important resource for people to survive in various fields but, at present, the problem of water pollution is becoming more and more serious. More and more scholars have paid attention to the problems of groundwater information collection and pollution monitoring [2]. With the gradual rise of the upsurge of developing and utilizing the ocean in various countries, more and more attention has been paid to the rights and interests of the ocean. Underwater Acoustic Sensor Networks (UASNs) have become a research focus [3].

UASNs are constructed by self-organizing sensor nodes distributed on the surface, underwater and water bottom. They have a wide range of application prospects in marine data collection, pollution monitoring, marine development, disaster prediction, auxiliary navigation and tactical monitoring and other sea-related fields [4,5]. In addition, to ensure the safety of underwater organisms and to enhance sensor networks, the system capacity deserves our attention [6,7]. Node localization technology is the premise of the application of UASNs. Only by knowing the location of the node, can the information collected be meaningful, so it is of great significance to study the node location technology of UASNs [8].

On land, we can monitor various environments through mobile data gathering schema in Wireless Sensor Networks [9] and these monitoring systems are relatively easy to deploy. However, due to the complex underwater environment, there are many challenges to the UASNs localization technology, such as the delay of signal transmission, a sensor deployed in the water will float with the flow of water and so on [10,11]. Therefore, many range-based algorithms will be affected by the environment under water and can not be well applied. The range-based algorithm will put forward higher requirements for ranging equipment and the power consumption and cost are also higher. On the other hand, at present, many range-based localization algorithms are designed according to the 2-Dimensional plane, whether it can be well applied to the 3-Dimensional space environment is also an unknown [12]. The range-free algorithm can realize autonomous localization without additional ranging equipment and it is not easy to be affected by the environment, so it can be well applied to UASNs localization.

When deploying UASNs, the stability of nodes is the first problem to be considered. Node deployment can be realized by the winch device proposed in the literature [12], and the sensor node can control itself by being equipped with its own air pump to avoid floating with the water flow as much as possible. In order to locate the nodes in UASNs, we first need some nodes to know their own location, and their location information can be obtained from the external GPS or through their own memory. These known nodes are called beacon nodes or anchor nodes and other nodes we call ordinary nodes or unknown nodes [13]. How to reduce the information storage capacity and improve transmission performance of underwater sensor networks is also worthy of our attention [14]. Zhang et al. proposed a recognition algorithm of D2D device for enhancing trust management of network communication [15]. The purpose of our UASNs localization is how to obtain the location information of the whole network unknown nodes through fewer beacon nodes.

The compressive sensing theory proposed in recent years has brought new opportunities for node localization in UASNs. The compressive sensing theory points out that as long as the original signal can be sparsely represented in some domains, the approximate and accurate reconstruction of the original signal can be realized by a small number of random linear observations [16,17]. By applying the compressive sensing to the UASNs localization, the problem that the information processing capacity of the node is insufficient is solved, only a small number of beacon nodes is needed and all the unknown node location can be obtained. In addition, because the energy of the node is limited and the computing power is not strong, the signal reconstruction process is generally carried out in a special information fusion center. In this way, the capability requirement of sensor node is reduced, which makes it cheap and simple. The theory of compressive sensing can also promote the development of WSNs in the further while relieving the pressure of WSNs sensor node.

In this paper, we mainly studied UASNs node localization technology. We hope to obtain more accurate node location information at lower cost and power consumption. The main contribution of this paper is the application of compressive sensing theory to UASNs. At the same time, the cost and energy consumption will be reduced and the localization accuracy will be improved. Besides, in order to solve the problem that the hops between nodes is only increased by integers in the localization algorithm, a hop correction algorithm is proposed, which further improves the localization performance. Compared with the traditional range-free algorithm, the simulation results show that the compressive sensing node localization algorithm based on hop number correction has the best localization performance under the same beacon node proportion, the same communication radius and the same total node number. At present, most practical applications still use range-based algorithm, which puts forward high requirements for cost and power consumption. The range-free algorithm we study does not need additional hardware support, and it is hoped that it will be helpful for underwater environment monitoring in the future.

The other chapters of this article are arranged as follows. The Section 2 describes the related work. In the Section 3, the compressive sensing localization algorithm is explained in detail. The Section 4 introduces the improved location algorithm. In the Section 5, the simulation results are summarized and analyzed. The Section 6 summarizes the full text and puts forward a new prospect for the future work.

## 2. Related Work

For the sensor network node localization algorithm, it is mainly divided into range-based and range-free. The node localization range-based algorithm needs to measure some arrival time information [18], angle information [19], signal indication strength information [20] and so on. The range-free algorithm only needs the connected information between the nodes to achieve self-localization. Typical range-free algorithms are Diffusion [21], LSVM [22], DV-hop [23] and so forth. Generally speaking, the node localization range-based algorithm has high accuracy but the cost is also high, and it is easily affected by the environment. Although the accuracy of range-free algorithm is relatively low, it can still meet the needs of most scenes, so it is of great practical significance to study the localization algorithm of range-free.

**Figure 1:**
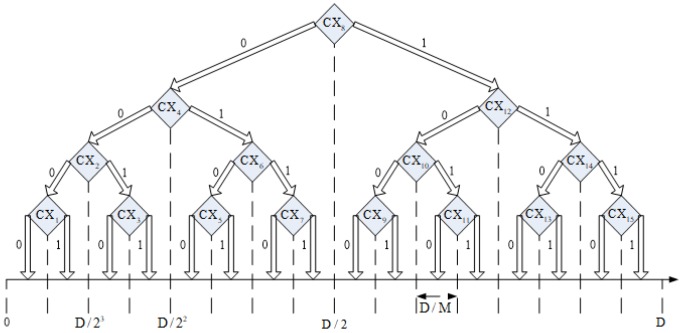
Binary tree classification.

Duc and Thinh [22] proposed a LSVM localization algorithm, which relies on the principle of classification of support vector machine(SVM) to complete node self-localization, as shown in Figure 1. The classifiers at the nodes of each binary tree use SVM classifiers, and the node x axis and y axis coordinates are divided into two and estimated separately.

**Figure 2:**
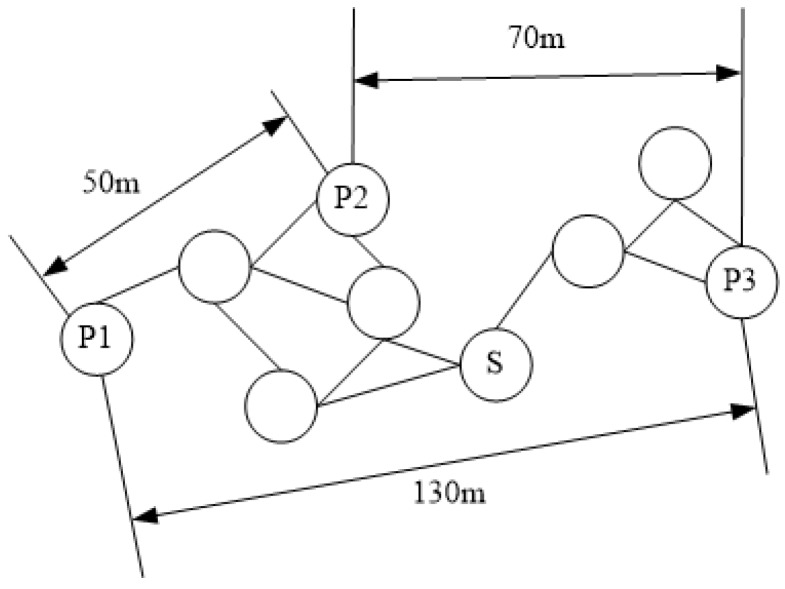
DV-hop algorithm.

The DV-Hop algorithm [23], as shown in Figure 2, uses a distance vector switching protocol to enable all nodes to obtain hop number information to beacon nodes, where beacon nodes use the information they obtain on the position and hop of other beacon nodes to estimate the average distance per hop in the network and broadcast it in the network through a controllable flooding protocol. In this way, most unknown node in the network can only receive information from the nearest beacon node, according to which the unknown node can estimate the distance between the beacon nodes. When the unknown node obtains information on at least three distances, its position can be estimated by the trilateral measurement method. Assuming P1, P2 and P3 are beacon nodes in the network, and S is the unknown node to be estimated, the S position can be determined by P1, P2 and P3.

At present, for the special underwater environment, it is a hot research topic to use underwater acoustic sensor network to monitor it. Therefore, many scholars are devoted to node localization of underwater acoustic sensor network. How to improve the localization accuracy is the focus of underwater acoustic sensor network node localization algorithm under the condition of low cost and power consumption.

First of all, some scholars use intelligent algorithm to optimize the performance of wireless sensor networks. Wang [24] et al. proposed an enhanced power efficiency gathering algorithm to improve the energy efficiency of the network. In References [25,26], they also put forward some clustering methods to save energy routing and improve data gathering.

Although these algorithms are proposed in wireless sensor networks, we expect them to be applied to underwater sensor networks in the future. In addition, in order to improve localization accuracy and reduce energy consumption, some 2-dimensional compressive sensing algorithms can still be applied to UASNs.

Zhao [27] et al. proposed a node localization algorithm based on compressive sensing in wireless sensor networks. The compressive sensing node localization algorithm estimates the position of the node by the combination of the compressive sensing and the centroid algorithm, which fully shows the spatial correlation among the nodes so that the algorithm can show good localization performance. In addition, because the sampling dictionary is composed of sampling atoms of each beacon node itself and is compressed, it will reduce the energy consumption of each node in the network and then reduce the communication consumption of the whole network.

He [28] et al. proposed a target location algorithm based on compressive sensing. In order to satisfy the orthogonality of the sensing matrix and not change the sparsity of the original signal, the sensing matrix is decomposed by LU and a more accurate target position is obtained.

Zhao [29] et al. proposed a cooperative localization algorithm for multi-anchor node cooperative UASNs, which divides the whole location into four sub-processes. Simulation results show that the algorithm proposed in this paper can effectively improve the localization rate and reduce the localization error under the condition of low energy consumption.

Although these algorithms are all proposed for 2-dimensional wireless sensor networks, they still have great inspiration for the localization of UASNs. Underwater sensor network localization has become a hot issue, many scholars have studied the localization algorithm of UASNs.

Based on the background of 2-Dimensional underwater sensor networks, Fazel et al. [30] proposed an improved ALOHA scheme based on compression perception, which can greatly reduce the influence of packet collision loss on information integrity in ALOHA protocol. However, the standardized dense deployment of sensor nodes considered in this scheme is often difficult to implement in the marine environment.

Liu et al. [31] proposed a virtual node aided localization algorithm for UASNs. The algorithm is divided into virtual node assisted static(VAS) localization algorithm and virtual node assisted dynamic(VAD) localization algorithm, which effectively improves localization accuracy and coverage.

Duing to TDOA algorithm could not be applied well for underwater complex environment, Yu et al. [32] proposed an improved AUV-based UASN time difference localization algorithm. The signal is sent regularly by AUV, which can be regarded as a series of virtual anchor nodes to complete the localization. The computational complexity of the algorithm is small and the accuracy is high.

Lin et al. [33] proposed a mobile node localization in UASNs. Firstly, the underwater monitoring area is divided into cubic modules. Through the energy relationship between the mobile node and the unknown node, the location of the unknown node is determined by the QR decomposition algorithm and the mobile path of the mobile node is also studied.

Many existing localization algorithms only focus on localization accuracy, neglecting the power consumption and data processing energy of the network. Based on the compressive sensing theory, this paper aims to realize the accurate localization of UASNs nodes with low network cost and power consumption. Specifically, we propose an UASNs node localization algorithm based on compressive sensing. According to the connected information between nodes, we can locate all unknown nodes through a small number of beacon nodes. In addition, in order to solve the problem that most of the hops of the algorithm are integers at this stage, we have modified it to further improve the localization performance. By comparing the localization errors of different beacon ratios, different communication radius and different total node number, the simulation results show that the compressive sensing algorithms modified based on hops have the best localization performance. The specific algorithm and performance are compared as follows.

## 3. Correlation Theory and Algorithm based on Compressive Sensing Node Localization

### 3.1. Compressive Sensing Theory

If the signal has a sparse representation in a transform domain, the signal is projected onto a low-dimensional space using a perceptual matrix (which is not associated with the transform domain and the dimension is much lower than the number of the signal number) to obtain an observed value (comprising a sufficient sample value for reconstructing the signal). And finally, the original signal is reconstructed from the high probability of the observation value by solving the optimal problem. As a result, the compressive sensing theory mainly includes the sparse representation of the signal, the design of the perception (observation) matrix and the design of the reconstruction algorithm. In the CS model, the signal *f* is sparse transformed, as shown in the following equation:

(1) $$f=\sum_{i=1}^{N}\varphi_{i}u_{i}=\Psi u$$

where μ, *f* are vectors and Ψ is a sparse matrix. The observed value *y* of the signal *f* is then obtained by the observation matrix a as follows:

(2) y=ΦΨu=Au

were *y* is the observation vector and Φ is the observation matrix. The sparse solution problem described above can be expressed as the following formula:

(3) $$\min_{u}{\left||u|\right|}_{0},\;\;\;\;s.t.\;\;\;\;Au=y$$

Finally, we can get the coefficient solution a by transforming it into an optimization problem.

### 3.2. Centroid Algorithm

The main idea of centroid algorithm is that unknown nodes take the geometric centroid of all beacon nodes in their communication range as their estimated positions. Its expression is shown in the following formula:

(4) $$\left(x,y,z\right)=\frac{\sum_{n=1}^{N}\left(x_{n},y_{n},z_{n}\right)}{N}$$

where $\left(x,y,z\right)$ is the estimated position of the ordinary node, $\left(x_{n},y_{n},z_{n}\right)$ is the position of the *n*-th beacon node and *N* is the total number of beacon nodes sensed by the node. In this paper, we locate the coordinates of ordinary nodes by weighted centroid algorithm.

When the position of nodes is estimated in UASNs, the compressive sensing algorithm relies on connectivity information to complete the localization, which can avoid complex ranging equipment and underwater ranging will have large errors due to transmission delay and so on. The compressive sensing algorithm can realize the localization of ordinary nodes through a small number of beacon nodes and can reduce the energy consumption and cost of UASNs. In the process of localization, when calculating the influence of beacon nodes on the weights of ordinary nodes, the position of beacon nodes is not used but only the connectivity information between nodes is used, so even if there are a few beacon nodes whose position is incorrect, it does not affect the sampling atoms obtained by beacon nodes, which will not affect the location of most target nodes. By combining with the centroid method, the spatial correlation between the target node and the beacon node is fully reflected and the localization accuracy of the algorithm is improved. The detailed algorithm is described below.

### 3.3. Underwater Acoustic Sensor Networks Model

**Figure 3:**
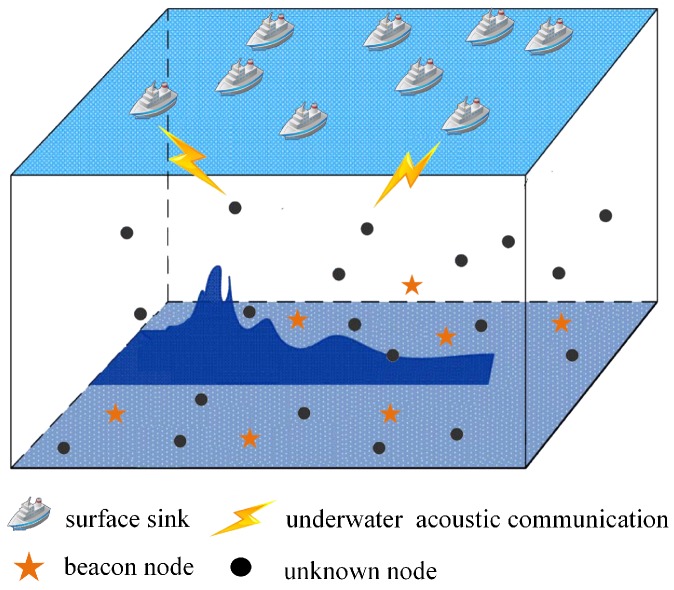
Underwater Acoustic Sensor Network Model.

We set it in the area to be monitored under water [0,X]×[0,Y]×[0,Z](X,Y,Z>0), as shown in Figure 3. Assuming there are *N* sensor nodes, and using $M_{1},M_{2},\cdots ,M_{N}$ indicates that the location of the *l* (*l* < *N*) nodes is known in advance, which is usually called beacons and the remaining *N*-*l* nodes are called unknown nodes. We assume that the communication radius of the sensor node is *R* and when both nodes are within the communication radius *R* of each other, they can communicate directly and the number of hops between them is 1. We take $h\left(M_{i},M_{j}\right)\left(i,j=1,2,\cdots ,N\right)$ to represent the minimum number of hops between node $M_{i}$ and $M_{j}$. Here we set up *l* (*l* < *N*) beacons, all of which know their location in advance and can communicate with each other. Now we want to locate the remaining *N*-*l* nodes through this *l* beacon node, without the help of additional ranging equipment and directly through the connected information between the nodes. Some existing range-free algorithms require directly communication between individual nodes or some beacon nodes within the communication range of all unknown nodes. In our algorithm, we only need unknown nodes to communicate with beacon nodes, whether they are single-hop or multi-hop, which greatly reduces the requirements of UASNs and is more suitable for underwater node localization.

### 3.4. Principle of Localization Algorithm

Suppose $\left(x\left(M_{i}\right),y\left(M_{i}\right),z\left(M_{i}\right)\right)$ is the coordinate position of the *l*-th beacon node. $\varphi_{i}={\left(h\left(S_{i},S_{1}\right),h\left(S_{i},S_{2}\right),\ldots ,h\left(S_{i},S_{l}\right)\right)}^{T}\in {R^{l}}^{\times 1}\left(i=1,2,\ldots ,l\right)$ is the hop number information between the *i*-th beacon node and all *l* beacon nodes, where $h(S_{i},Si)=0$, that is, the connectivity information from the node to itself is 0. We will use a sparse transformation matrix Ψ to represent the number of hops between anchor nodes:

(5) $$\Psi ={\left[\varphi_{1},\varphi_{2},\cdot \cdot \cdot ,\varphi_{l}\right]}^{T}\in R^{l\times l}$$

Similarly, we use $f_{j}={\left(h\left(S_{j},S_{1}\right),h\left(S_{j},S_{2}\right),\ldots ,h\left(S_{j},S_{l}\right)\right)}^{T}\left(j=N-l+1,\ldots ,N\right)$ represents the hop number information for the *j*-th ordinary node and all l anchor nodes. According to the sparse transformation basis $\Psi ,f_{j}$ can be sparse decomposition into:

(6) $$f_{j}=\sum_{i=1}^{l}\varphi_{i}\mu_{j,i}=\Psi \mu_{j}$$

where $\mu_{j}={\left({\mu_{j,}}_{1},\ldots ,\mu_{j,i},\ldots ,\mu_{j,k}\right)}^{T}$ is a column vector, $\mu_{j,i}$ is the degree of correlation between the *j*-th unknown node and the *i*-th anchor node under sparse transformation basis. Generally speaking, the closer the two nodes are to the UASNs, the larger their correlation coefficients are. When the two nodes are far away, the correlation coefficients will be very small or even close to 0. In addition, for a ordinary node, it may be represented by several closer beacon nodes and their correlation coefficients will be large. On the contrary, the correlation coefficient of beacon nodes far away from unknown nodes will be very small or approximately regarded as 0. It can also be understood that $\mu_{j}$ is sparse. Therefore, we can reconstruct these correlation coefficients by using CS theory.

Therefore, the CS sensing matrix *A* and the observed $y_{j}$ can be expressed as follows:

(7) A=ΦΨ

(8) $$y_{j}=\Phi f_{j}=\Phi \Psi \mu_{j}=A\mu_{j}$$

where *A* can be represented by Gaussian matrix. By compressive sensing algorithm, we can accurately reconstruct $\mu_{j}$ and use $\mu_{j}$ to get the weight of the *i*-th beacon node to the *j*-th ordinary node, the expression is as follows:

(9) $$\omega_{j,i}=\mu_{j,i}/\sum_{i=1}^{k}\mu_{j,i}$$

Finally, the estimated position $(x\left(S_{j}\right),y\left(S_{j}\right),z\left(S_{j}\right)$ of the *j*-th ordinary node can be obtained by the weighted centroid algorithm [34].

(10) $$\left(x\left(S_{j}\right),y\left(S_{j}\right),z\left(S_{j}\right)\right)=\sum_{i=1}^{l}\omega_{i,j}\left(x\left(S_{i}\right),y\left(S_{i}\right),z\left(S_{i}\right)\right)$$

**Figure 4:**
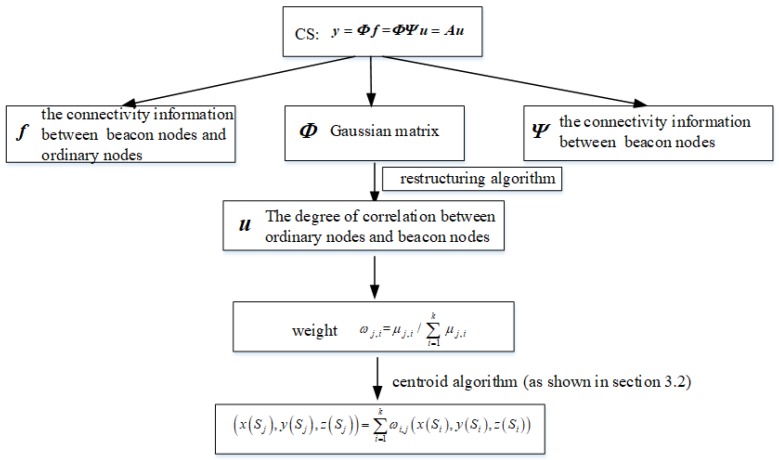
Relationships between various terms.

In order to show our algorithm more intuitively, I will use the following drawing to illustrate relationships between various terms. Figure 4 shows the relationships between various terms in the compressive sensing algorithm directly and clearly.

The connected information collected by the algorithm based on compressive sensing is the minimum number of hops from each node to the beacon node, and they are all integers, so that the connected information of the more adjacent nodes is very similar. Therefore, a sparse transform base composed of connected information of all beacon nodes may be not accurate enough, when the connected information of the target node is decomposed. For example, there are two beacon nodes close to the target node and within the communication radius of the target node. We think that the number of hops between them is 1, so that the correlation degree of the beacon node closest to the target node may not be the maximum. Therefore, the sparse decomposition obtained by the traditional hop number method is certainly not optimal, so the localization accuracy of the target node obtained by it will also be affected.

## 4. Compressive Sensing Node Localization Algorithm Based on Hops Correction

### 4.1. Improved Principle

In the compressive sensing node localization algorithm, LSVM algorithm and DV-Hop algorithm, because the hop from each node to the beacon node can only be an integer, the relationship between the number of hops between each node and their distance will not be very accurate. So that the corresponding relationship between the connected relationship and the distance will not be accurate enough, thus affecting the accuracy of sparse decomposition. In order to solve the problem that the hop number is an integer in the traditional method, Xiao [35] provided a method for correcting the number of hops. In this paper, we can use this method to further improve the localization accuracy. In order to illustrate the improved algorithm in this paper, several parameters are defined at first.

**Definition** **1.** *The ratio of the actual distance between the two anchor nodes i and j to the communication radius R of all nodes is defined as the ideal hop number, represented by $H_{ij}$, that is,*

(11) $$H_{ij}=\frac{d_{ij}}{R}$$

**Definition** **2.** *The relative error between the actual hop number and the ideal hop number between nodes is defined as the deviation factor, which is represented by $\alpha_{ij}$, that is,*

(12) $$\alpha_{ij}=\frac{hops_{ij}-H_{ij}}{hops_{ij}}$$

*where $hops_{ij}$ is the actual hop number between anchor nodes i and j. The deviation factor reflects the extent to which each hop of the node deviates from the ideal hop count. The larger $\alpha_{ij}$ is, the greater the degree of deviation from the ideal hop number is and the greater the error is when the actual hop number is used instead of the ideal hop number.*

**Definition** **3.** *Assuming that the actual hop number between anchor node i and j is $hops_{ij}$ and the deviation factor is $\alpha_{ij}$, the correction coefficient $\omega_{ij}$ of hop number is defined as follows:*

(13) $$\omega_{ij}=1-{\alpha_{i}}_{j}$$

### 4.2. Number of Hops between Anchor Nodes

In order to make the actual hop number as close to the ideal value as possible, the number of hops between anchor nodes is modified by the following formula:

(14) $$hops{}_{ij}^{\prime }={\omega_{i}}_{j}hops_{ij}$$

where $hops{}_{ij}^{\prime }$ is the number of hops corrected between anchor nodes and $hops_{ij}$ is the actual number of hops between anchor nodes.

### 4.3. Correction of Hops between Unknown Nodes and Anchor Nodes

In the traditional location algorithm, the unknown nodes to beacon nodes are integer hops and the actual hops are not all integers. In order to solve this problem, the hop number between unknown node and anchor node is modified in this paper.

#### 4.3.1. Number of Hops from an Unknown Node to Its Nearest Anchor Node

Suppose that the actual hop number between unknown section *P* and anchor node *l* is $hops_{pi}$ and the number of anchor nodes is *l*. Using formula (15) to corrects it and the modified hop number is as follows:

(15) $$h_{pi}=\frac{\sum_{i\neq j}^{l}\omega_{ij}}{l-1}hops_{pi}$$

where $hops_{pi}$ is the actual hop number between unknown node and anchor node *i* and $\omega_{ij}$ calculated by formula (13). $\sum_{i\neq j}^{l}\omega_{ij}/\left(l-1\right)$ is the average value of the deviation factor between the nearest anchor node *l* and other anchor nodes, which makes full use of the hop number information of each anchor node and can better reflect the characteristics of the node in the whole network.

#### 4.3.2. Number of Hops between Unknown Nodes and Other Anchor Nodes

When the unknown node *P* calculates the number of hops from other anchor nodes, the formula (16) is used to correct the number of hops from other anchor nodes:

(16) $$h_{pj}=\omega_{ij}hops_{pj}$$

where $\omega_{ij}$ is obtained by formula (12) and $hops_{pi}$ is the actual number of hops between unknown nodes and other anchor nodes. In this way, the traditional method is improved to solve the inaccurate problem and the localization performance is further improved.

Based on the above principle, the number of hops from each node to its neighbor node can be obtained, and then the connected information from each node to all beacon nodes can be obtained by using these modified hops. Because the connectivity information is obtained by modifying the hop number, the connectivity information from each node to all beacon nodes will be very accurate. We bring the new hop information into the algorithm flow of Section 3.3 and we can get a more accurate node position.

## 5. Simulation Analysis

### 5.1. Effectiveness Analysis of Compressive Sensing Algorithm

**Figure 5:**
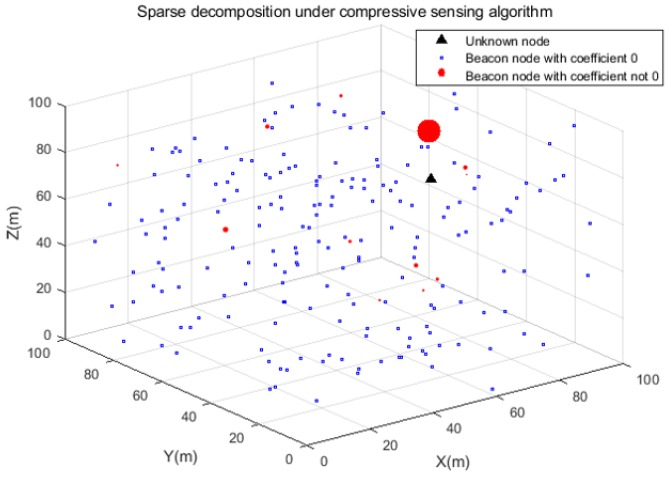
Sparse decomposition under compressive sensing algorithm.

In a given underwater monitoring area of 100 m × 100 m × 100 m, we randomly distribute 1000 sensor nodes. Among them, we assume that there are 200 beacon nodes, the rest are unknown nodes to be measured and the communication radius of all nodes is 20 m. We assume that all nodes can communicate with each other and that fluctuations with the current can be ignored. For any unknown node, we can give a schematic diagram of the correlation coefficient between it and the beacon node, as shown in Figure 5.

As we can see from Figure 5, for any unknown node, only a few beacon nodes have a correlation coefficient of non-zero and most of the correlation coefficients are zero. In other words, the correlation coefficients are sparse and the closer the two nodes are, the larger their correlation coefficients are, so we can use compressive sensing theory to find out the position of unknown nodes through a small number of beacon nodes.

### 5.2. Compression Ratio Analysis

**Figure 6:**
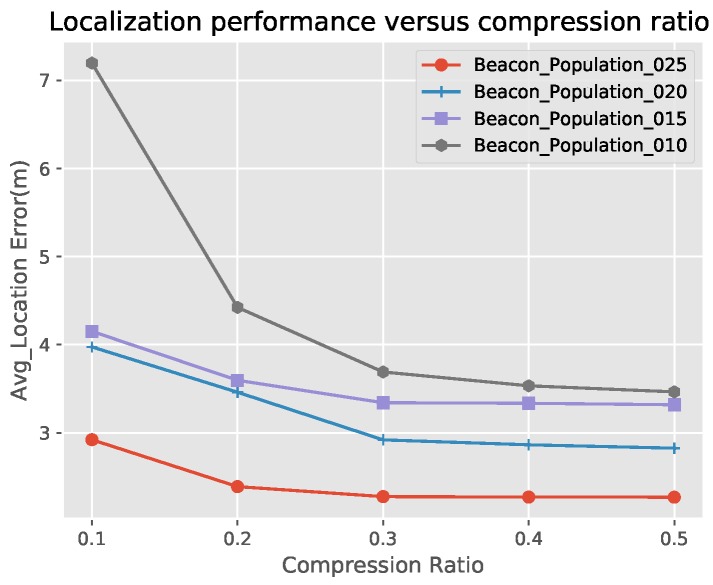
Compression ratio analysis.

In UASNs, the consumption of energy is also an issue of concern to us. In the compressive sensing algorithm used in this paper, we use the Gaussian matrix as the measurement matrix and we can see the curve of the localization error varies with the compression ratio by simulation. We assume that there are 1000 nodes, the communication radius is 30m and the proportion of anchor nodes is 0.1, 0.15, 0.2, 0.25. From Figure 6, we can see that larger compression ratio will reduce our localization error but when the compression ratio reaches a certain value, the localization error tends to be stable and the larger compression ratio will consume more energy, so we should choose the appropriate compression ratio value.

### 5.3. The Relationship between the Localization Error and the Proportion of Beacon Nodes

**Figure 7:**
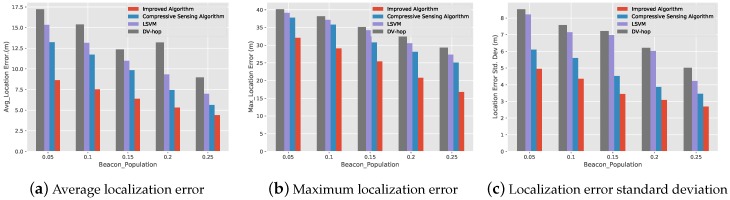
The relationship diagram between the localization error and the proportion of beacon nodes.

Figure 7 shows the relationship between the localization performance of the improved compressive sensing algorithm, compressive sensing algorithm, LSVM algorithm and DV-Hop algorithm with the proportion of beacon nodes when the communication radius is 25 at 1000 sensor nodes. a, b and c is the schematic diagram of the average localization error, the maximum localization error and the standard deviation of the localization error varies the proportion of beacon nodes for 4 algorithms. From Figure 7, we can see that with the increase of the proportion of beacon nodes, the 4 algorithms localization error of both algorithms is decreasing. No matter what the proportion of beacon nodes is, our improved algorithm is superior to the other three algorithms. This is due to the improved algorithm, which uses the hop number correction method to improve the connected information, so that the connected information from each node to the beacon node will be able to accurately describe the spatial relationship between them. So the target node will be more accurately described by the beacon node, thus further improving the performance of the algorithm.

### 5.4. The Relationship between Localization Performance and Communication Radius

**Figure 8:**
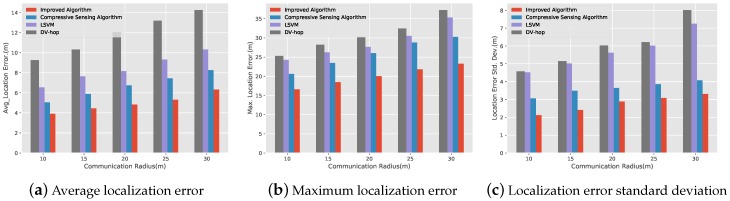
The relationship diagram between localization performance and communication radius.

Figure 8 shows 4 algorithms relationship between localization performance and communication radius under the condition that 1000 sensor nodes and the proportion of beacon nodes is 0.2. As can be seen from Figure 8a, the improved algorithm has smaller average localization error compared with the compressive sensing algorithm and the other two algorithms. It is also easy to see from Figure 8b,c that the improved algorithm has smaller maximum localization error and standard deviation than the other three algorithms. We can also see that with the increase of communication radius, the localization error is increasing. Although a larger communication radius is more conducive to node communication, we should also consider it comprehensively when selecting the node communication radius.

### 5.5. The Relationship Diagram between Localization Performance and Total Node Number

**Figure 9:**
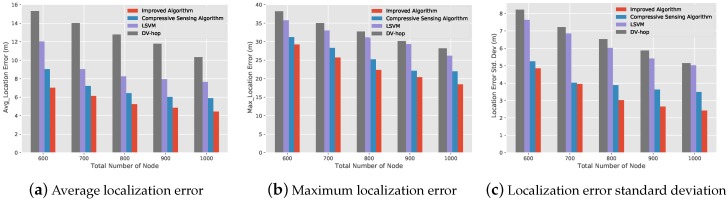
The relationship diagram between localization performance and total number of node.

Figure 9 shows 4 algorithms relationship between localization performance and the total number of node under the condition that the proportion of beacon nodes is 0.2 and the communication radius is 15 m. As can be seen from Figure 9a, compared with the compressive sensing algorithm and the other two algorithms, the improved algorithm has the smallest average localization error. It is also easy to see from Figure 9b, c that the improved algorithm has smaller maximum localization error and standard deviation than the other three algorithms. We can also see that under the condition that the proportion of beacon nodes remains unchanged, the larger the total number of node and the smaller the localization error. More nodes can better ensure communication between nodes but also better reflect the connected information relationship between nodes, but more nodes need more cost and energy. Therefore, in practical application, we should choose the number of nodes according to the specific scenario.

## 6. Conclusions

The underwater environment is complex and many sensor network node localization technologies at this stage can not be well applied. In this paper, the node localization algorithm of sensor network based on compressive sensing is applied to underwater sensor network localization, which not only ensures the localization accuracy but also does not need additional cost and power consumption. In view of the shortcomings of the traditional hop number solving method, this paper also proposes a hop number correction algorithm. The simulation results show that the modified algorithm based on hops has better localization performance than the traditional algorithm under the same communication radius, the proportion of beacon nodes and the same total node number. The algorithm proposed in this paper hopes to be helpful to the future underwater environment monitoring. In the future, we will consider the mobile node localization of UASNs.
